# MicroRNA-15a modulates lens epithelial cells apoptosis and proliferation through targeting B-cell lymphoma-2 and E2F transcription factor 3 in age-related cataracts

**DOI:** 10.1042/BSR20191773

**Published:** 2019-12-06

**Authors:** Qiao Li, HaiTao Pan, QingHuai Liu

**Affiliations:** 1 Department of Ophthalmology, The First Affiliated Hospital of Nanjing Medical University, Nanjing, China; 2 Department of Ophthalmology, The Affiliated Hospital of Xuzhou Medical University, Xuzhou, China; 3 Department of Cadre Health Care, Jinling Hospital, School of Medicine, Nanjing University, Nanjing 210002, Jiangsu Province, China

**Keywords:** age-related cataract, BCL2, E2F3, miR-15a

## Abstract

Age-related cataract remains a serious problem in the aged over the world. MicroRNAs are abnormally expressed in various diseases including age-related cataract. MicroRNA-15a (MicroRNA-15a) has been involved in various diseases and plays crucial roles in many cellular processes. However, the mechanism of microRNA-15a in the genesis of cataract remains barely known. We therefore aimed to investigate the role of microRNA-15a in the cataract. Herein, human lens epithelial B3 cells, HLE-B3 cells were treated with 200 μmol/l H_2_O_2_ for 24 h. H_2_O_2_ was utilized in our study to induce HLE-B3 cells injury. We observed that cell apoptosis was induced by the treatment of H_2_O_2_ and meanwhile, cell proliferation was repressed by 200 μmol/l H_2_O_2_. Then, it was found that microRNA-15a was significantly increased with the H_2_O_2_ exposure *in vitro*. Importantly, B-cell lymphoma-2 (BCL2) and E2F transcription factor 3 (E2F3) exert crucial roles in cell apoptosis and cell proliferation. We found that BCL2 and E2F3 were greatly reduced by 200 μmol/l H_2_O_2_ in human lens epithelial cells. In addition, microRNA-15a overexpression induced cell apoptosis and repressed cell proliferation through suppressing BCL2 and E2F3. Subsequently, BCL2 and E2F3 were predicted as a direct target of microRNA-15a. The direct correlation between microRNA-15a and BCL2/E2F3 was confirmed by dual luciferase reporter assay. In conclusion, we demonstrated that microRNA-15a triggered apoptosis and repressed the proliferation of HLE-B3 cells by modulating BCL2 and E2F3.

## Introduction

Cataract has a high incidence all over the world and it can contribute to the blinding eye disease [1–3]. Numerous factors can lead to the development of cataract. But age and ocular tissue degeneration are the most common inducements. Age-related cataract can affect 46% of the 180 million visually disabled people [4,5]. Hence, identifying the effective biomarkers of cataract can reduce cataract incidence and blinding rates.

As well established, lens epithelial cell apoptosis is an early event in cataract progression [6]. Damages, such as oxidative stress to the epithelial cells can contribute a lot to age-related cataract [7]. Lens epithelial cell apoptosis induced by oxidative stress is a common cellular basis in cataract [8].

MicroRNAs are a small non-protein coding RNAs with 20–25 nucleotides, which can regulate gene expression post-transcriptionally [9]. MicroRNAs regulate mRNA degradation or translation through combining with their targeting mRNAs [10]. So far, microRNAs are involved in a variety of cell processes including proliferation, migration and apoptosis [11–13]. It has been reported that in many studies abnormal expression of miRNAs is closely associated with the pathogenesis of many age-related diseases, including cataract progression [14–16]. For example, miR-34a can promote mitochondrial dysfunction-triggered apoptosis in human lens epithelial cells via targeting Notch2 [17]. miR-26a and -26b inhibit lens fibrosis and cataract through regulating Jagged-1/Notch signaling pathway [18]. In addition, let-7b induces lens epithelial cell apoptosis by targeting Lgr4 [19].

In our current study, we focused on the biological role of microRNA-15a in age-related cataract. We aimed to in vestigate the biological role of microRNA-15a in age-related cataract and the mechanisms of action. Therefore, we demonstrated that microRNA-15a modulated age-related cataract progression through targeting B-cell lymphoma-2 (BCL2) and E2F transcription factor 3 (E2F3) *in vitro*.

## Materials and methods

### Cell culture

Human lens epithelial B3 (HLE-B3) cells were purchased from American Type Culture Collection (ATCC; Rockville, MD, U.S.A.). Cells were cultured in minimum essential medium (MEM; Gibco, Carlsbad, CA, U.S.A.) added with 10% fetal bovine serum (FBS; Gibco, Carlsbad, CA, U.S.A.) in a humidified chamber with 5% CO_2_.

### Cell transfection

MicroRNA-15a mimics or their parental negative controls (RiboBio, Nanjing, China) were transfected into the cells using Lipofectamine 2000 reagent (Invitrogen, Carlsbad, CA, U.S.A.).

### Cell counting kit-8 assay

Cells were seeded in a 96-well plate for a whole night. Then, 10 μl Cell Counting Kit-8 (CCK-8) reagents (Dojindo Molecular Technologies, Tokyo, Japan) were added to the cells and the cells were incubated for 4 h. A microplate reader (Bio-Tek, Winooski, VT, U.S.A.) was utilized to test the absorbance at 450 nm.

### 5-ethynyl-2′-deoxyuridine assay

To detect the function of microRNA-15a on cell proliferation, 5-ethynyl-2′-deoxyuridine (EdU) proliferation assay (RiboBio, Nanjing, China) was conducted. After transfection for 48 h, cells were incubated with 50 μM EdU. An Apollo and DAPI staining were employed to detect the EdU-positive cells.

### Apoptosis detection

Cell apoptosis was assessed using an Annexin V-FITC/PI apoptosis detection kit. Briefly, 1  ×  10^4^ cells were grown in six-well plates per well. Afterward, cells were digested using trypsin without EDTA, harvested and washed three times using PBS diluted in 500 μl Annexin binding buffer. For each sample, 5 μl Annexin-V-FITC and 5 μl propidium iodide were added to cell suspension and then the cells were incubated for 15 min in the dark. Cell apoptosis was evaluated by quantifying the Annexin V-FITC-positive cells. Subsequently, flow cytometry data were plotted and analyzed using the fluorescence-activated cell sorting (FACS-Vantage) system and Cell Ouest software (Becton-Dickinson, San Jose, CA, U.S.A.).

### Caspase-3 activity assay

Caspase-3 activity was detected using a caspase-3 assay kit (Abcam, Cambridge, U.K.). Cells were lysed in 50 μl chilled cell lysis buffer. A total of 50 μl cell lysis buffer containing 100 μg protein was added, 50 μl of 2× reaction buffer, 0.5 μl of 10 mmol/l dl-dithiothreitol (DTT) and 5 μl caspase-3 catalytic substrate DEVD-pNA substrate were used. After incubation, the absorbance in each well was tested at 405 nm using a microplate ELISA reader.

### Western blot analysis

Cell extracts were prepared using RIPA buffer containing protein inhibitor cocktail (Roche, Penzberg, Germany). Samples were separated on sodium dodecyl sulfate/polyacrylamide gel electrophoresis (SDS/PAGE) and electroblotted on to PVDF membranes. Then, the membranes were blocked using 5% skim milk in TBS-T buffer at room temperature for 1 h. The membrane was incubated with rabbit monoclonal antibody against human BCL2 and E2F3 (Abcam, Cambridge, U.K.) followed by horseradish peroxidase (HRP)-labeled goat anti-rabbit IgG (Southern Biotech, AL, U.S.A.). The protein bands were exposed with the ECL chemiluminescence kit (Pierce, Rockford, IL, U.S.A.).

### Quantitative real-time PCR

The RNAiso Plus (TaKaRaBio Technology, Dalian, China) was used to extract RNA. RNA reverse transcription was carried out using Prime Script™ RT Master Mix and qPCR was performed using SYBR Premix ExTaq II (TaKaRa Bio Technology, Dalian, China). Primers for Quantitative real-time PCR (qRT-PCR) were displayed in Table 1. Applied Biosystems 7900 Real-Time PCR System (Applied Biosystems, Foster City, CA, U.S.A.) was used.

**Table 1:** Primers for real-time PCR

Genes	Forward (5′–3′)	Reverse (5′–3′)
*GAPDH*	AAGAAGGTGGTGAAGCAGGC	GTCAAAGGTGGAGGAGTGGG
*U6*	CTCGCTTCGGCAGCACATA	CAGTGCAGGGTCCGAGGTA
*microRNA-15a*	CGCCTAGCAGCACATAATGG	AGTGCAGGGTCCGAGGTAT
*BCL2*	CTGCACCTGACGCCCTTCACC	CACATGACCCCACCGAACTCAAAGA
*E2F3*	CGGTCTGCTCACCAAGAAGT	CCTCTTCTGCACCTTGAGCA

### Luciferase activity assay

The wild-type (WT) or mutant (MUT) BCL2/E2F3 binding microRNA-15a was subcloned into pGL3 basic vector (Promega, Madison, WI, U.S.A.). microRNA-15a mimics (RiboBio, Guangzhou, China) were co-transfected with 10 μg pLUC-WT-BCL2/E2F3 or pLUC-MUT-BCL2/E2F3 into the cells.

### Statistical analysis

Statistical data analysis was carried out using GraphPad Prism 6.0 (GraphPad Software, Inc., La Jolla, CA) statistical packages. Each experiment was performed in triplicate and data were presented as mean ± SD. Statistical analysis was performed using Student’s *t* test or one-way analysis of variance. Statistical significance was considered when *P*-value was less than 0.05.

## Results

### H_2_O_2_ induced apoptosis and repressed proliferation in human lens epithelial cells

**Figure 1:**
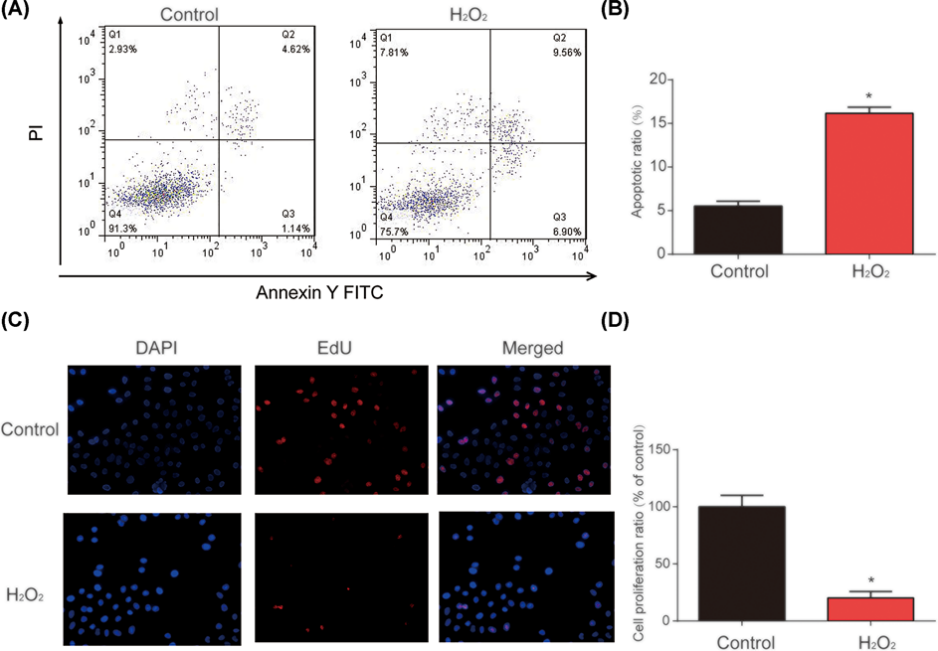
H_2_O_2_ induced apoptosis and inhibited proliferation in HLE-B3 cells (**A,B**) Flow cytometry analysis of the apoptosis induced by H_2_O_2_. Cells were treated with 200 μmol/l H_2_O_2_ for 24 h. Flow cytometry was performed to test cell apoptosis. (**C,D**) Analysis of the proliferation induced by H_2_O_2_. EDU assay was performed to test cell proliferation. Three independent experiments were carried out. Error bars stand for the mean ± SD of at least triplicate experiments. *P<0.05.

First, as shown in Figure 1A,B, HLE-B3 cell apoptosis was increased by 200 μmol/l H_2_O_2_ for 24 h. In addition, we observed that HLE-B3 cell proliferation was significantly inhibited by 200 μmol/l H_2_O_2_ *in vitro* (Figure 1C,D). These findings indicated that H_2_O_2_ induced apoptosis and depressed proliferation in HLE-B3.

### H_2_O_2_ up-regulated microRNA-15a and down-regulated BCL2, E2F3 in HLE-B3 cells

**Figure 2:**
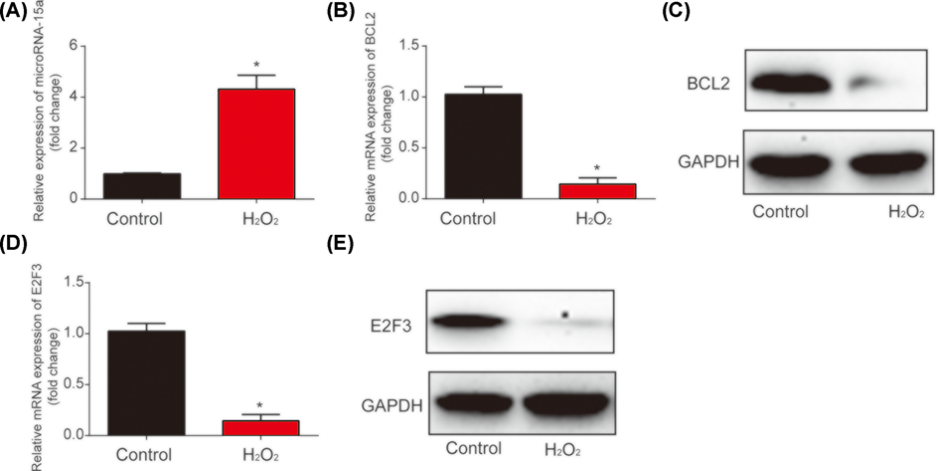
Expression of microRNA-15a, BCL2 and E2F3 in HLE-B3 cells incubated with 200 μmol/l H_2_O_2_ (**A**) MicroRNA-15a expression in HLE-B3 cells. Cells were indicated with 200 μmol/l H_2_O_2_ for 24 h. (**B**) BCL2 mRNA expression in HLE-B3 cells. (**C**) BCL2 protein expression in HLE-B3 cells. (**D**) E2F3 mRNA expression in HLE-B3 cells. (**E**) E2F3 protein expression in HLE-B3 cells. Three independent experiments were carried out. Error bars stand for the mean ± SD of at least triplicate experiments. *P<0.05.

Then, a significant increase in microRNA-15a was observed in H_2_O_2_-treated HLE-B3 cells compared with the control group (Figure 2A). Additionally, BCL2 mRNA and protein expression was obviously decreased in HLE-B3 cells incubated with 200 μmol/l H_2_O_2_ (Figure 2B,C). In addition, E2F3 mRNA and protein expression was also greatly decreased by H_2_O_2_ *in vitro* (Figure 2D,E). These implied that microRNA-15a/BCL2/E2F3 was involved in cataract development.

### MicroRNA-15a regulated human lens epithelial cell apoptosis and cell proliferation

**Figure 3:**
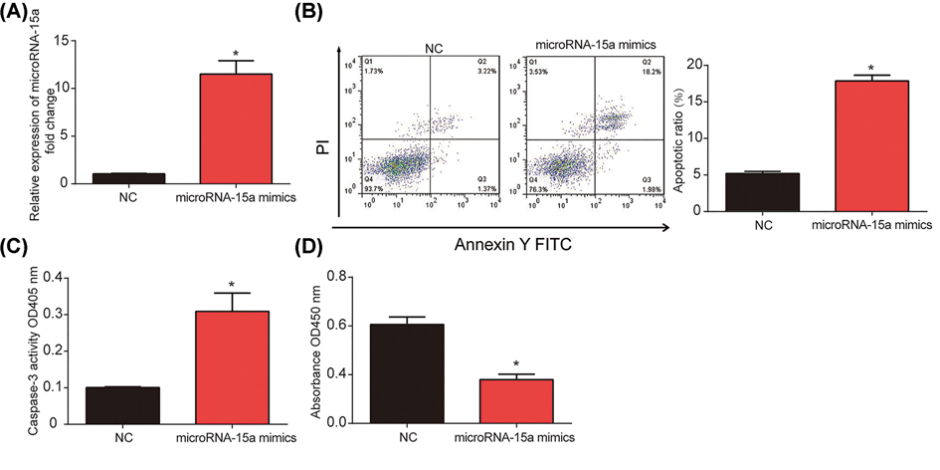
MicroRNA-15a regulated human lens epithelial cell proliferation and apoptosis (**A**) MicroRNA-15a expression in HLE-B3 cells. Cells were transfected with microRNA-15a mimics for 48 h. (**B**) Flow cytometry analysis of the apoptosis in HLE-B3 cells. (**C**) Caspase-3 activity in HLE-B3 cells. (**D**) Cell viability measured by the CCK-8 assay in HLE-B3 cells. Three independent experiments were carried out. Error bars stand for the mean ± SD of at least triplicate experiments. *P<0.05.

Then, further investigation was conducted to explore the effect of microRNA-15a on apoptosis and proliferation. HLE-B3 cells were transfected with microRNA-15a mimics or mimic controls for 48 h. As shown in Figure 3A, microRNA-15a was significantly increased by microRNA-15a mimics in HLE-B3 cells. Subsequently, flow cytometry assay indicated that overexpression of microRNA-15a induced cell apoptosis (Figure 3B). In addition, caspase-3 activity assay showed that microRNA-15a mimic group significantly elevated caspase-3 activity (Figure 3C). Next, CCK-8 assay was carried out and it was found that cell viability was repressed by microRNA-15a mimics in Figure 3D. These further revealed that microRNA-15a regulated the proliferation and apoptosis of human lens epithelial cells.

### MicroRNA-15a regulated BCL2 and E2F3 expression in human lens epithelial cells

**Figure 4:**
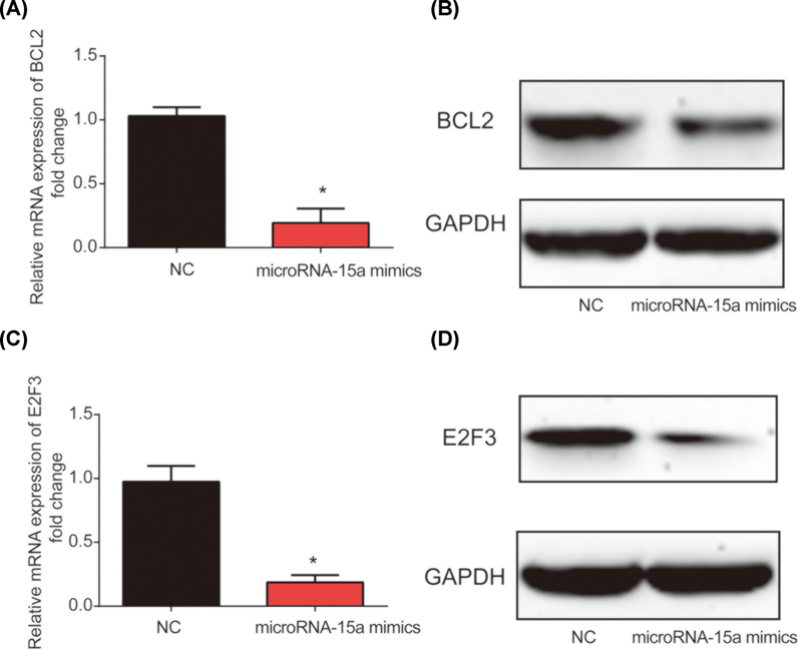
BCL2 and E2F3 expressions were inhibited by overexpression of microRNA-15a in HLE-B3 cells (**A**) Quantitative RT-PCR data of BCL2 mRNA in HLE-B3 cells. (**B**) Western blot data of protein levels of BCL-2 in HLE-B3 cells. (**C**) E2F3 mRNA expression in HLE-B3 cells. (**D**) Protein levels of E2F3 in HLE-B3 cells. Three independent experiments were carried out. Error bars stand for the mean ± SD of at least triplicate experiments. *P<0.05.

After HLE-B3 cells were transfected with microRNA-15a mimics or mimic controls for 48 h, BCL2 and E2F3 mRNA expression and protein expression were detected. As compared with the mimic control group, BCL2 expression were significantly decreased in microRNA-15a mimics group (Figure 4A,B). In addition, E2F3 mRNA (Figure 4C) and protein (Figure 4D) levels were also significantly reduced compared with the control group. These data suggested that microRNA-15a regulated BCL2 and E2F3 expression in HLE-B3 cells.

### BCL2 and E2F3 were the targets of microRNA-15a

**Figure 5:**
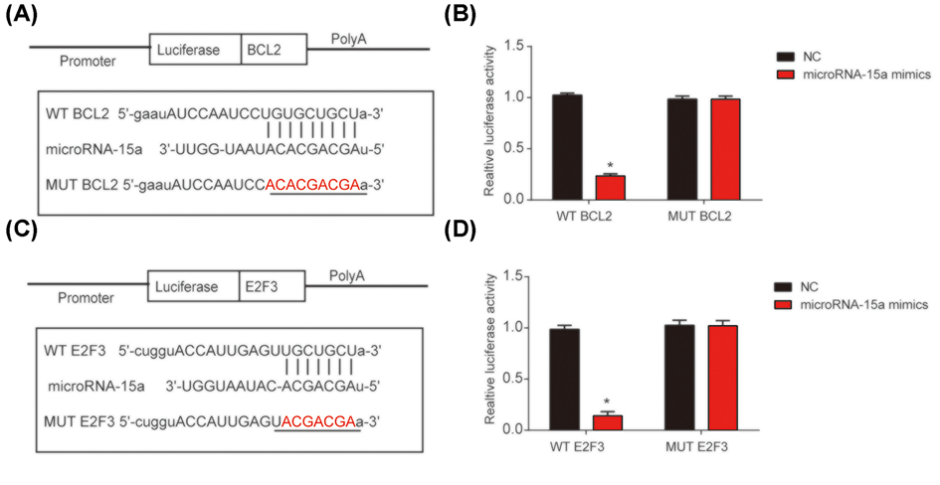
BCL2 and E2F3 were direct targets of microRNA-15a (**A**) The luciferase reporter constructs containing the WT-BCL2 or MUT-BCL2 sequence. (**B**) WT-BCL2 or MUT-BCL2 was co-transfected into HLE-B3 cells with microRNA-15a mimics or their corresponding negative controls. (**C**) The luciferase reporter constructs containing the wild-type (WT-E2F3) or mutant E2F3 (MUT-E2F3) sequence. (**D**) WT-E2F3 or MUT-E2F3 was co-transfected into HLE-B3 cells with microRNA-15a mimics or their corresponding negative controls. Three independent experiments were carried out. Error bars stand for the mean ± SD of at least triplicate experiments. *P<0.05.

To investigate the direct target gene of microRNA-15a, bioinformatics analysis (TargetScan, Starbase, miRanda and miRDB database) was performed. BCL2 and E2F3 were predicted as the direct targets of microRNA-15a. Binding regions between microRNA-15a and BCL2 was shown in Figure 5A and the Luciferase reporter plasmids of WT-BCL2 and MUT-BCL2 binding sites were displayed. Co-transfection of the luciferase reporter plasmid containing the WT with microRNA-15a mimics decreased the reporter activity in HLE-B3 cells (Figure 5B). In addition, the Luciferase reporter plasmids of WT E2F3 and mutant E2F3 binding sites were exhibited in Figure 5C. Consistently, co-transfection of the luciferase reporter plasmid containing the WT with microRNA-15a mimics also suppressed the reporter activity (Figure 5D). The results showed that BCL2 and E2F3 directly targeted microRNA-15a.

## Discussion

MicroRNAs are involved in various pathological processes including cell growth and apoptosis and they can also represent a potential target for the diagnosis, prevention and treatment of age-related cataracts [20–22]. In our study, we observed microRNA-15a was greatly up-regulated in HLE-B3 cells indicated with 200 μmol/l H_2_O_2_. Meanwhile, BCL2 and E2F3 was obviously down-regulated by 200 μmol/l H_2_O_2_ in HLE-B3 cells. Overexpression of microRNA-15a was able to inhibit cell proliferation and induce cell apoptosis by targeting BCL2 and E2F3. Moreover, the negative interaction between BCL2, E2F3 and microRNA-15a was proved in our study. A novel mechanism of microRNA-15a/BCL2/E2F3 axis in age-related cataracts was revealed in our present study.

Oxidants can trigger apoptosis and contribute to cataract development [23]. In our present research, we found that 200 μmol/l H_2_O_2_ induced apoptosis and inhibited cell proliferation of HLE-B3 significantly. miR-15 family are clustered on three separate chromosomes [24] and the miR-15 family can play a significant role in various cancers [25,26]. For instance, knockdown microRNA-15a promotes the development and induces the EMT process of NSCLC cells [27]. MicroRNA-15a inhibits endometrial cancer cell growth through Wnt/β-catenin signaling by repressing WNT3A [28]. Recently, microRNA-15a in age-related cataract patients is reported to be increased [29].

The BCL2 gene family and its related protein bcl-2 were the first apoptosis-related genes to be studied [30]. BCL2 family genes can play a regulatory role in apoptosis. For example, miR-34a can promote apoptosis of human lens epithelial cells through down-regulating BCL2 [31]. Using informatics analysis, we found that the famous anti-apoptotic gene BCL2 might act as a direct target of microRNA-15a.

The E2F transcription factor family contains eight members, which plays an important role in cellular proliferation, differentiation and apoptosis [32]. E2F1-3 transcription factors exert essential roles in cellular proliferation [33]. For example, miR-203a can suppress cell proliferation through targeting E2F3 in human gastric cancer [34]. MiR-217 inhibits pancreatic cancer cell proliferation via targeting E2F3 [35]. In addition, miR-34a can suppress proliferation and induce apoptosis of human lens epithelial cells through targeting E2F3 [36].

In summary, the results of our present study demonstrated that microRNA-15a was able to modulate lens epithelial cells apoptosis and proliferation through targeting BCL2 and E2F3 in age-related cataracts. Our observations can provide novel insights into the potential therapeutic applications for age-related cataracts treatment.

## Data Availability Statement

All data are available upon request.

## Abbreviations

BCL2: B-cell lymphoma-2
DAPI: 4′,6-diamidino-2-phenylindole
DEVD-pNA: Caspase-3 substrate (chromogenic)
EdU: 5-ethynyl-2′-deoxyuridine
E2F: encodes a family of transcription factors
E2F3: E2F transcription factor 3
HLE-B3: human lens epithelial B3
MUT: mutant
qPCR: quantitative polymerase chain reaction
RIPA: Radioimmunoprecipitation assay
TBS-T: tris-buffered saline (TBS) and Polysorbate 20
WNT3A: Wnt family member 3A
WT: wild-type
